# Application of Digital Olfaction for Table Olive Industry

**DOI:** 10.3390/s22155702

**Published:** 2022-07-29

**Authors:** Ramiro Sánchez, Antonio Fernández, Elisabet Martín-Tornero, Félix Meléndez, Jesús Lozano, Daniel Martín-Vertedor

**Affiliations:** 1 Technological Institute of Food and Agriculture CICYTEX-INTAEX, Junta of Extremadura, Avda. Adolfo Suárez s/n, 06007 Badajoz, Spain; ramiro.sanchez@juntaex.es; 2 Departamento de Química Analítica, Universidad de Extremadura, 06006 Badajoz, Spain; elisabetmt@unex.es; 3 Industrial Engineering School, University of Extremadura, 06006 Badajoz, Spain; felixmv@unex.es (F.M.); jesuslozano@unex.es (J.L.); 4 Research Institute of Agricultural Resources (INURA), Avda. de la Investigación s/n, Campus Universitario, 06071 Badajoz, Spain

**Keywords:** E-nose, digital olfaction, volatile compounds, sensory analysis, table olives

## Abstract

The International Olive Council (IOC) established that olives must be free of odors, off-flavors, and absent of abnormal ongoing alterations or fermentations. The use of electronic devices could help when classifying defects in a fast, non-destructive, cheap, and environmentally friendly way. For all of that, table olives were evaluated according to IOC regulation in order to classify the defect predominant perceiving (DPP) of the table olives and their intensity. Abnormal fermentation defects of Spanish-style table olives were assessed previously by an IOC-validated tasting panel. ‘Zapateria’, ‘Putrid’, and ‘Butyric’ were the defects found at different concentrations. Different volatile compounds were identified by gas chromatography in altered table olives. The same samples were measured with an electronic nose device (E-nose). E-nose data combined with chemometrics algorithms, such as PCA and PLS-DA, were able to successfully discriminate between healthy and non-healthy table olives, being this last one also separated between the first and second categories. Volatile compounds obtained with gas chromatography could be related to the E-nose measuring and sensory analysis, being capable of matching the different defects with their correspondents’ volatile compounds.

## 1. Introduction

Spain is the leading country in table olive production, being present in almost all countries such as Argentina, Peru, Portugal, Egypt, Morocco, and Turkey. The table olive sector is highly important to the agri-food industry due to job generation and production volume, transformation, commercialization, and exportation activities [1].

The most frequent elaboration style is “Spanish-style”, which consists in treating *Olea europea* spp. Fruits with caustic soda in order to eliminate bitterness. After that, table olives will be fermented for several in a NaCl solution. As the fermentation goes by, there are some critical points in months which abnormalities could happen, leading to defective table olives. These unexpected processes are one of the main causes of high economic loss for table olive producers.

Nowadays, only physical defects are categorized as extra, first, or second category olives [2]. Although, the absence of strange flavors, odors, or symptoms of abnormal fermentations or ongoing alterations is a compulsory statement present in the current regulation. For that reason, the performance of sensory analysis by a well-trained tasting panel and validated by IOC [3] should be accomplished for classification purposes. Nevertheless, the IOC protocol is not mandatory but only a recommendation. Certain olfactory defects such as zapateria, musty, butyric, rancid, or vinegary sensations should be classified by the sensory panel [4,5]. Zapateria defect is the predominant sensation perceived by the tasters when present in table olives. A combination of volatile fatty acids generated during abnormal fermentations is responsible for this particular rotten leather sensation, according to IOC. Musty or humidity defect generates mold smell. The butyric defect is a butter or cheese off-flavor. Finally, the putrid defect is responsible for the decaying organic matter odor. Bad industrial practices are the most usual causes of these defects, facilitating uncontrolled fermentation process development.

Chromatographic analysis of volatile compounds can also assist in identifying responsible compounds for abnormal fermentation [6,7,8,9]. However, sensory analysis based on an expert panel and volatile fraction characterization of fermented olives based on gas chromatography are laborious, costly, and time-consuming processes requiring complex equipment and/or qualified personnel. Therefore, it is relevant to develop a fast and reliable technique to differentiate table olives based on their organoleptic properties. Abnormal fermentations should be identified by this protocol in order to perceive the early defect and have the opportunity of controlling them.

Nowadays, electronic instrumentation is emerging to propose new ways of non-destructive, fast, low-cost, and environmentally friendly measurement. In that sense, the electronic nose (E-nose) is a powerful sensory device that is able to discriminate aroma profiles of different matrices [10,11,12,13]. This device has also been used to classify olives on olive trees [14]. In this respect, the E-nose may be an effective alternative to identify different types of abnormal fermentations in table olives on an industrial scale. Non-destructive E-nose could be complementary to the tasting panel. Early off-flavor identification can be useful to correct them before the olives become unacceptable and unmarketable.

The development of a functional E-nose methodology to discriminate between healthy and non-healthy samples (this last group is divided into first and second categories) of Spanish-style table olives according to their sensory attributes has been performed. Data were contrasted with gas chromatography and sensory analysis of the table olives by the testing panel. The novelty that this work is the portability and adaptation of the E-nose to the standard olive tasting cups, which allow the reproduction of the evaluation protocol of sensory attributes recommended by the IOC of which table olives can be classified into quality categories. Having an objective instrument to classify in categories accessible to the table olive industry could lead to the consequent improvement in table olives quality. Thus, the aim of this work was to develop a quality control methodology in the industry capable of discriminating between healthy and unhealthy samples, as well as classifying them into different categories according to their sensory attributes with a digital olfaction device.

## 2. Materials and Methods

### 2.1. Experimental Design

Olives (*Olea europaea* L.) of the “Carrasqueña” variety were obtained in a research field of the CICYTEX research center (Badajoz, Spain). Olives were harvested at the green stage of maturation during the 2021/2022 campaign. They were processed in a semi-scale station according to the Spanish-style protocol [15].

The product was introduced into a fermenter with the capacity of 236 L. Olives were submitted to lye treatment, and after all, the product was washed with water and immersed into small fermenters of 10 L of capacity with brine at 5.5% NaCl (Sigma-Aldrich, St. Louis, MO, USA). Spontaneous microbiological fermentation was carried out for 121 days [15]. Each week, the tasting panel in charge of the assay performed a sensory analysis to discriminate between olives without abnormal fermentations of the ones that have them. Samples that did not present a clear defect were discarded by the panelists. Thus, the number of samples previously selected by the tasting panel with natural microbiological alteration was 81.

**Figure 1:**
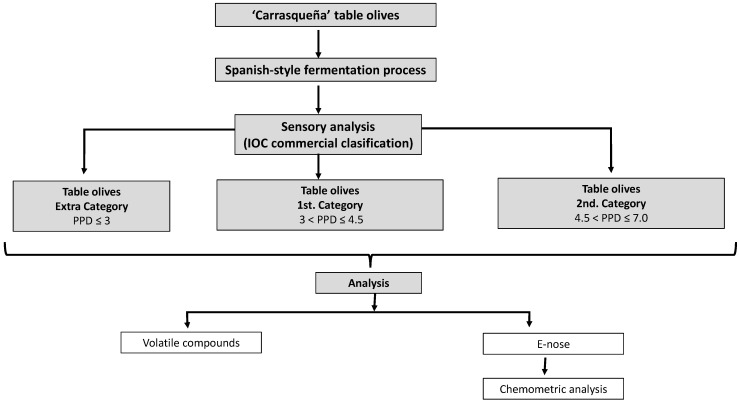
Diagram of the overall experiment design.

The samples were stored in refrigeration (4 °C) when the target defect was perceived until the analysis was performed. A sample with controlled fermentation was also stored (control). A diagram of the overall experimental design is shown in Figure 1.

### 2.2. Analyses

#### 2.2.1. Sensory Analysis

The variety used for this assay was ‘Carrasqueña’, a typical Spanish table olive. These samples were analyzed by a well-trained sensory panel from CICYTEX-INTAEX Research Center (Extremadura, Spain). A standard glass jar was used to place table olives inside in at the same height, filling them with 10 mL of brine. On a well-defined scale from 1 to 11, abnormal fermentations perceived by the panelists were evaluated in terms of intensity and off-odor perception. The results were displayed as mean defect values, considering them acceptable when the variation coefficient was less than 20. Finally, table olives were categorized according to the quality classification elaborated by the IOC [3]. A total of 81 samples were studied.

For the statistical analysis, a one-way ANOVA was performed plus Tukey’s multiple range test to establish statistically significant differences between the different samples. Significance was set at *p* < 0.05. The software used was SPSS 18.0 (SPSS Inc., Chicago, IL, USA). Data were expressed as mean and standard deviations (SD).

#### 2.2.2. Analysis of Volatile Compounds

A triple quadrupole gas chromatograph (Scion 456-GC, Bruker, Madrid, Spain) followed the method described by López-López et al. [7] and was used to analyze volatile compounds by triplicate. SPME was used to sample from the headspace (40 °C for 15 min) with a polydimethylsiloxane/divinylbenzene (PDMS/DVB) StableFlex fiber (65 μm, Supelco, Madrid, Spain). Once desorption occurred at the injection port of the gas chromatograph (250 °C for 15 min), the components were divided using a capillary column (30 m × 0.25 mm, ID: 0.25 mm, VF5MS, Agilent, Madrid, Spain). NIST 2.0 MS library was used to analytes identification through mass fragmentation comparative analysis.

#### 2.2.3. E-Nose Analysis

The E-nose used in this work was designed by the University of Extremadura and consisted of an array of 11 metal oxide (MOX) sensors. These sensors are distributed on four chips previously explained in previous research [13]. The microprocessor receives the signal from the sensors, processes them, and sends the data to a smartphone via Bluetooth. A response value of the sensor array is obtained every second. Thus, to characterize the sensor response curves, the formula of the maximum signal value minus the minimum signal value multiplied by 100 and subtracted by: (MAX − MIN) × 100 − 1) was used. As a result, a data vector with 11 rows (sensors) was obtained for each sample. Finally, they are transferred to the computer for chemometric analysis. The measurements and data obtaining with the electronic devise are shown in https://susy.mdpi.com/user/submission/video/5f6c41e532625088e8bf165b38a5ce18.

This device described in more detail by Arroyo et al. [13] has low power consumption and is the size of a hockey puck, making it easy to transport. This array of sensors has been used previously by the authors [13,16,17]. The measurements with the E-nose were performed following the same protocol as the table olive tasting panel that is based on IOC recommendations [3]. Three olives with about 10 mL of brine were placed in standard tasting glasses, covered with a watch glass, and placed in a block thermostat at 25 °C. One tasting cup remained empty. Each data acquisition cycle consisted of two parts, one adsorption, and one desorption. Alternately, the E-nose was placed in the cups with samples and air. First, 30 s in the cup with air to achieve desorption of volatiles on the sensors and return the signal to baseline. Next, the E-nose measured the volatiles in the headspace of the samples for 60 s, and the signals from the sensors were recorded.

#### 2.2.4. Multivariate Data Analysis

E-nose data were first analyzed using principal component analysis (PCA) to perform exploratory analysis. This algorithm minimizes the dimensionality of the variables to a smaller number, denominated principal components. The aim of this unsupervised method was to detect outliers and recognize patterns or cluster formation. As the variables were measured in different units, the original variables were autoscaled. Subsequently, the supervised classification analysis called partial least squares discriminant analysis (PLS-DA) [18] was applied to build a classification model. This algorithm identifies the components or latent variables (LV) that most discriminate between the different groups of samples. These variables were selected by cross-validation with the leave-one-out procedure. A supervised classification requires prior knowledge of the class of each sample, and that was obtained with the previous classification of the tasting panel. Specifically, a model was developed to discriminate between table olive samples belonging to three different categories. The confusion matrix of the model was constructed to represent the prediction success. The proportion of the correct predictions was calculated from the sum of the diagonal elements found in the confusion matrices.

Data analysis was performed using Matlab R2016b version 9.1 (The Mathworks Inc., Natick, MA, USA) with PLS_Toolbox 8.2.1 (Eigenvector Research Inc., Wenatchee, WA, USA).

## 3. Results

First, the organoleptic profile and the volatile compounds of the samples studied are described. Next, the results of E-nose technology are shown to distinguish between table olives’ health and those with different defects. Finally, an experimental model was developed with the data provided with E-nose to classify olives according to their quality.

### 3.1. Sensory Analysis of Spanish-Style Table Olives

Spanish-style table olives were sensorially classified into different categories according to the defect predominant perceived (DPP) by the tasting panel (Table 1). Table olives samples without any sensory alteration were classified as the “Extra” category. However, other samples showed several defects that are related to abnormal fermentation. The first batch of samples had a defect concentration range of 3.8–4.1. The main defects described in different samples by panelists were ‘Zapateria’, ‘Butyric’, and ‘Putrid’. These olives were classified in the first commercial category (3 < DPP ≤ 4.5). As can be seen, other samples presented a range of defects between 5.9 and 6.2 being the DPP ‘Zapateria’ and ‘Putrid’. These olives were classified into a second category (4.5 < DPP ≤ 7.0).

### 3.2. Volatile Profile of Spanish-Style Table Olives

Table olives altered with different types and intensities of defects were analyzed to determine the volatile compounds profile (Table 2). Different volatile compounds were identified in altered table olives. The main volatiles compounds in healthy table olives were creosol (48.1%), 2-Ethenyl-1,1-dimethyl-3-methylene-cyclohexane (18.0%), acetic acid (9.6%), phenylethyl alcohol (9.7%), and benzoic acid (8.6%). Nevertheless, some of these volatile compounds decreased considerably in altered olives. That is the case of acetic acid, creosol, or benzoic acid, whose concentration decreases, especially in olives with a high intensity of defect. Regardless of the intensity of the defect found, other different compounds appear. The main constituents of ‘Zapateria’ samples were butanoic acid (14.6–22.8%), propylene glycol (9.6–15.9%), (E)-3-hexenoic acid (8.2–14.4%), hexanoic acid (1.8–5.5%), cyclohexanecarboxylic acid (1.8–7.4%) and pentanoic acid (valeric acid) (3.2–4.5%). The ‘Putrid’ defect also presented a particular volatile profile. The major constituent present in table olives were propanoic acid (17.2–23.4%), isopropyl alcohol (17.2–21.3%), 2,4-dimethyl-heptane (15.4–17.9%), and phenylethyl alcohol (12.0–19.6%). The volatile compounds responsible for the ‘Butyric’ defect were butanoic acid (40.7%), pentanoic acid (11.8%), propanoic acid (3.6%), and butan-2-ol (4.6%). These compounds presented a higher content in samples classified in the second category.

On the other hand, it should be noted that certain volatile compounds identified are only detected in some of the defects studied. It is the case of (Z)-3-hexen-1-ol and 2-ethenyl-1,1-dimethyl-3-methylene-cyclohexane in healthy olives, propylene glycol, or (E)-3-hexenoic acid in ‘Zapateria’ table olives, isopropyl alcohol in ‘Putrid’ olives or butan-2-ol in ‘Butyric’ table olives.

**Figure 2:**
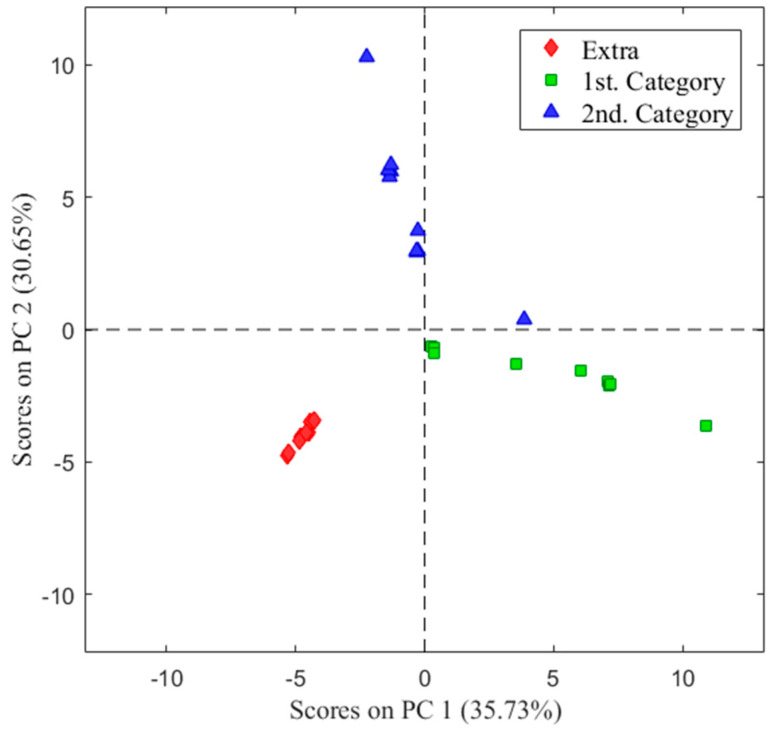
Score plot of the principal component analysis (PCA) analysis for healthy (extra) and defective olives (1st and 2nd categories).

A principal component analysis (PCA) was performed to observe the interaction of the variables and grouping of the samples originated from the profile data of table olive analysis (Figure 2). The total variance of data was explained a 35.73% by PC1 and 30.65% by PC2.

A clear sample differentiation according to their olfactory characteristics was shown by the two components-based models. It can be seen that the exploratory analysis was able to discriminate healthy olives from those with a defect in the fermentation process.

### 3.3. E-Nose Classification of Spanish-Style Table Olives

**Figure 3:**
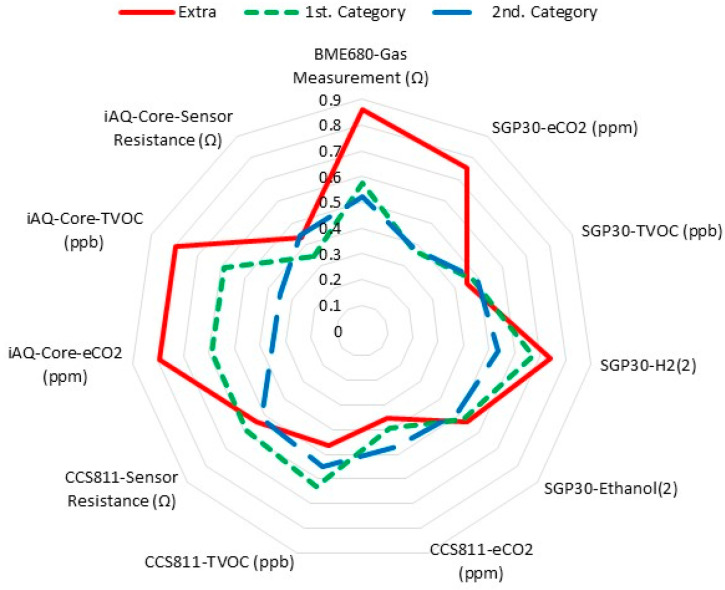
Radial plots of the sensor’s responses to Spanish-style table olives of different categories.

The E-nose was used to classify the table olives previously analyzed by the tasting panel. The olives classified into three different categories were placed in the tasting cups. The E-nose was placed on top of the cups to evaluate the headspace of the samples. The E-nose consists of 11 sensors that emit an electrical signal in the presence of volatile compounds in the samples. The response of each sensor to the same sample is different, so we can describe an olfactory pattern with the combination of the 11 signals. To represent the response patterns of the sensors to the extra, first, and second categories, a radial plot was drawn (Figure 3). For it, the data of E-nose obtained for each category were first normalized according to the formula (X_i_ − X_MIN_)/(X_MAX_ − X_MIN_); where X_i_ is the experimental value measured for sample i; X_MIN_ is the minimum experimental value of the data series and X_MAX_ is the maximum experimental value of the data series. Afterward, the average values of the data series for each category were obtained, and finally, they were represented in the radial graph. It can be clearly seen in Figure 3 that the amplitude of the signals from extra table olives is generally of greater magnitude than the signals from olives classified in the first and second categories. The curves representing the categories are clearly different from each other.

**Figure 4:**
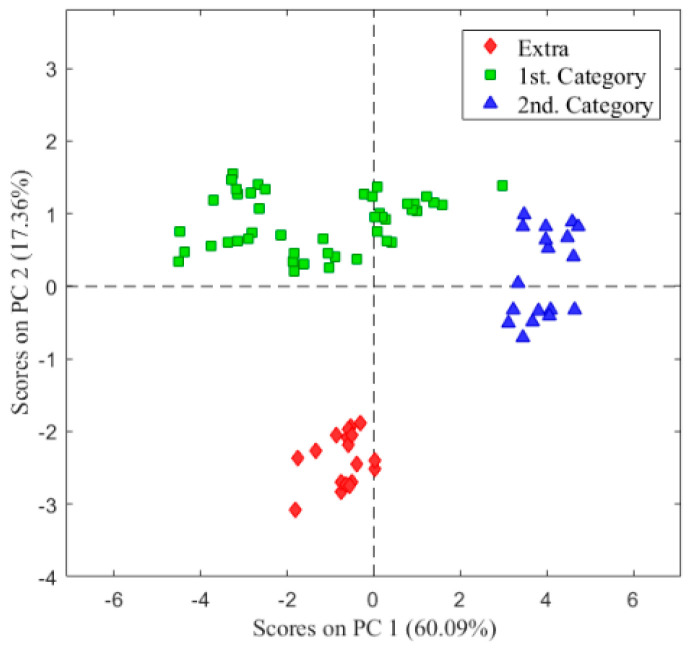
Score plot of the principal component analysis (PCA) analysis for table olives classified in different categories.

The E-nose data of table olive samples from the three categories were first analyzed by principal component analysis (PCA). Scores values of the two first principal components are plotted against each other in Figure 4. It can be observed by grouping the samples into three categories. The first two principal components are enough to explain 77% of the total variance of the data. There is clear discrimination between extra olives and the first and second categories.

Following the revealing results obtained in the PCA, a supervised classification analysis was performed using PLS-DA and leave-one-out cross-validation. The results are shown in Table 3 as a confusion matrix, where the diagonal represents the number of samples that have been correctly assigned. As we can see, all the samples were correctly classified in their category, being the success rate of the classification model of 100%. The 100% correct classification result is consistent with the clear difference in aroma of the sensory analysis samples of each category. The extra category has a DPP value of 0, and the first one has a minimum difference with the extra of 3.8 points and 1.8 with the second category. Therefore, the E-nose can be a useful tool to develop a classification and quality control methodology accessible for routine use in any industry, capable of discriminating between healthy and unhealthy samples, as well as classifying them into different categories according to their sensory attributes.

## 4. Discussion

When the chemical conditions are not appropriate during the elaboration process of Spanish-style table olives, undesirable alterations occur. This was the case in this study when olives were fermented with low concentrations of salt after lye and washing treatments. Thus, different kinds and intensities of defects in Spanish-style table olives were found. Table olives were sensory analyzed by a tasting panel following the methodology established by the International Olive Council [3]. When industrialists realize that certain batches of olives show alterations, the intensity of the defect is usually too high. This causes olives to have defects that, depending on their intensity, make the product belong to one commercial category or another, which could negatively affect the business economy. Normally, samples are taken from fermenters with possible abnormal fermentation and should be analyzed by a professional tasting panel to assess their quality. Industrial should control the chemical composition of the fermenters to avoid alterations. The lack of monitoring of the product during olives fermentation causes a pH modification, which can contribute to abnormal fermentation. In contrast, some fermenters studied presented any kind of alterations that make olives present a characteristic aromatic pattern. Some of these samples were classified into the extra category (DPP < 3), although they did not present sensory defects. Note that these are olives of the highest commercial category. On the other hand, tasters detected some negative attributes in olives that were analyzed by the defect predominant perceived (DPP). Some groups of samples were classified into the first category because the DPP was higher than 3 and less than or equal to 4.5. ‘Zapateria’, ‘Butyric’, and ‘Putrid’ were the main defects found in these samples. Furthermore, tasters indicated some defects such as ‘Zapateria’ and ‘Putrid’ in high intensity. Thus, these olives were classified in the second category because the DPP was higher than 4.5 and less than or equal to 7.0 (Table 1). We have to highlight that the olives studied could be legally marketed despite the significant sensory alterations [3,19]. Therefore, these olives that were not controlled during fermentation presented anomalous fermentations that caused a marked sensory profile with defects with different intensities. In this sense, researchers [20] assessed table olives from different companies by a tasting panel detecting different intensity defects such as butyric, putrid, zapateria, musty, or winey–vinegary.

In the same way, Spanish-style table olives, after finishing the fermentation process, present particular volatile compounds and non-volatile aromatic compounds that contribute to the sensory aroma [21]. These olives were classified as ‘Extra’ category presented volatile organic compounds (VOCs) responsible for positive aromas. Creosol, acetic acid, or 2-ethenyl-1,1-dimethyl-3-methylene-cyclohexane are the main VOCs presented in healthy olives. Other alcohols compound such as (Z)-3-hexen-1-ol or phenylethyl alcohol are products formed in alcoholic fermentations [22], presenting a fruity fragrance to apples or bananas [23]. These aromatic compounds also appear in altered olives but in lower concentrations. Defected olives are characterized by an increase in certain volatile compounds characteristic of the defect. That is the case of propylene glycol, 2,4-hexadienoic acid, methyl ester, or (E)-3-hexenoic acid in ‘Zapateria’ alteration, or isopropyl alcohol in ‘Putrid’ defect, and butan-2-ol in ‘Butyric’. These outcomes are in agreement with previous studies [8,24,25]. Cyclohexanecarboxylic acid also appears in the ‘Zapateria’ defect at low concentrations, but it has been identified as a key compound of this alteration in some studies [25]. Other researchers [26] indicated that this compound, in combination with other VOCs, is responsible for the characteristic unpleasant odor in the ‘Zapateria’ defect. Butanoic acid, pentanoic acid, propanoic acid, and butan-2-ol are VOCs presented in ‘Butyric’ and ‘Zapateria’ defect and propanoic acid even in ‘Putrid’ defect. These carboxylic acids are associated with cheesy odor (propanoic acid) and buttery and cheesy odor (butanoic acid) [27]. This result is in agreement with previous studies [25]. Furthermore, certain molds generate undesirable products during Spanish-style elaboration processes [28], producing musty aromas. In general, the VOCs of the samples studied allowed to classify the samples according to their commercial category. Clearly, the PCA of the VOCs classified olives according to the intensity and the type of defect, which play an important role in their commercial classification.

On the other hand, the radial profile of the response of each sensor of the E-nose to the aromatic profile of the olives shows that each sensor reacts in a certain way to the VOCs of the headspace of the samples. The radial figure was different from one commercial category to another. Next, the PCA model was performed to discriminate table olives categories with the E-nose. This electronic device was able to discriminate olives based on their commercial category. This discrimination could be due to the particular aroma profiles of the VOCs present in the different samples. E-nose was even able to discriminate samples with different defects in each category. Few references exist on the discrimination of olives submitted to abnormal fermentation using an E-nose. Researchers have discriminated by E-nose Spanish-style table olives inoculated with different altering [29] and those with abnormal fermentation with sensory defects to zapateria, butyric and putrid [28]. Other researchers presented an E-nose system able to discriminate different fungal species [30,31]. Other researchers have also established a PLS-DA model in order to produce a predictive classification model capable of separating the table olives varieties [32] and table olives stuffed with flavored hydrocolloids [33,34] that can be used as a rapid and inexpensive screening method for discrimination of standard characteristic aroma in Spanish-style and Californian-style table olives. All in all, the outcomes presented in our research prove that this electronic device is a powerful tool to discriminate VOCs produced by Spanish-style table olives. This equipment could be easily implemented in the table olive processing industry due to its installation simplicity, small size, fast measurement, precision, and low cost of analysis.

## 5. Conclusions

Spanish-style table olives fermented under uncontrolled chemical conditions showed characteristic alterations in aroma and intensity. ‘Zapateria’, ‘Butyric’, and ‘Putrid’ sensory alterations were the main defect perceived by tasters being classified in different commercial categories according to the criteria set by the IOC. These olives presented a volatile compounds profile characteristic that made the E-nose successfully used for discriminating olives from different commercial categories. Thus, this device could be useful for detecting olfactory sensations provoked by abnormal fermentation. The commercial classification made for E-nose matched the results obtained by the panelists. Therefore, this electronic device is complementary to the panel test and, combined with chemometric analysis, can be used to perform rapid, inexpensive, non-destructive, and environmentally friendly qualitative analysis for the table olives industry. Installing this device in the industry would be an effective strategy to detect incipient alterations during the fermentation process. Therefore, table olives quality could be improved thanks to these emergent electronic olfactive technologies. With this study, it has been put into account that a simple design can incorporate electronic devices as an alternative to conventional methods to assure food quality during its industrial processing, avoiding important economic losses.

## Figures and Tables

**Table 1:** Defect predominantly perceived (DPP) of Spanish-style table olive. Different lowercase letters mean a statistically significant difference between altered olives within the same category (one-way ANOVA followed by Tukey’s test, *p* < 0.05). Different uppercase letters mean a statistically significant difference between each altered olive in different categories (one-way ANOVA followed by Tukey’s test, *p* < 0.05).

	Extra	1st Category			2nd Category	
	DPP ≤ 3	3 < DPP ≤ 4.5			4.5 < DPP ≤ 7.0	
Sensory evaluation	‘No defect’	‘Zapateria’	‘Putrid’	‘Butyric’	‘Zapateria’	‘Putrid’
	n.d. *n* = 18	4.1 ± 0.9 ns A *n* = 15	3.8 ± 0.7 ns A *n* = 15	3.9 ± 0.8 ns *n* = 15	6.2 ± 0.8 ns B *n* = 9	5.9 ± 0.9 ns B *n* = 9

n.d., not detected; ns: not significant differences.

**Table 2:** Relative percentage of volatile compounds obtained from altered Spanish-style table olives classified into different commercial categories. RT, retention time.

RT (min)	Volatile Compounds	Extra	1st. Category			2nd. Category	
			‘Zapateria’	‘Putrid’	‘Butyric’	‘Zapateria’	‘Putrid’
1.8	Isopropyl alcohol			17.2 ± 1.3 *			21.3 ± 4.2 *
2.4	Butan-2-ol				4.5 ± 0.6 *		
2.7	Acetic acid	9.6 ± 1.5	8.3 ± 1.2	5.4 ± 0.7	7.5 ± 1.2	1.9 ± 0.5	
4.8	2-methyl-butan-1-ol			5.2 ± 0.8			6.5 ± 2.2
4.9	Propanoic acid		0.9 ± 0.2	17.2 ± 7.4	3.6 ± 0.8	2.6 ± 0.6	23.4 ± 8.7
5.8	Propylene glycol		9.6 ± 1.1 *			15.9 ± 5.8 *	
6.7	2,4-dimethyl-heptane	1.3 ± 0.5		15.4 ± 5.7			17.9 ± 8.6
8.2	Butanoic acid		14.6 ± 4.6		40.7 ± 8.4	22.8 ± 9.5	
9.7	(Z)-3-Hexen-1-ol	0.7 ± 0.1 *					
11.2	Styrene						
13.5	Pentanoic acid		3.2 ± 0.6		11.8 ± 2.7	4.5 ± 2.4	
17.8	2,4-Hexadienoic acid, methyl ester		3.2 ± 0.7 *			9.6 ± 3.7 *	
18.5	Hexanoic acid		1.8 ± 0.6 *			5.5 ± 1.1 *	
20.7	(E)-3-Hexenoic acid		8.2 ± 1.2 *			14.4 ± 6.7 *	
21.9	2-methoxy-phenol	4.0 ± 0.4	2.6 ± 0.7			0.8 ± 0.2	4.1 ± 0.6
22.0	2,4-Hexadienoic acid, ethyl ester		3.1 ± 0.6 *			9.5 ± 2.1 *	
23.0	2-Ethenyl-1,1-dimethyl-3-methylene-cyclohexane	18.0 ± 2.6 *					
23.3	Phenylethyl Alcohol	9.7 ± 1.5		12.0 ± 4,4			19.6 ± 8.9
26.5	Cyclohexanecarboxylic acid		1.8 ± 0.4 *			7.4 ± 2.1 *	
27.0	Creosol	48.1 ± 6.8	35.2 ± 9.4	27.5 ± 9.6	25.4 ± 10.4	3.2 ± 0.5	7.2 ± 0.8
28.2	Benzoic acid	8.6 ± 1.3	7.5 ± 5.9		6.5 ± 3.6	1.9 ± 0.4	

*: Compound unique to a concrete defect; empty cells correspond to non-detected measures.

**Table 3:** Confusion matrix obtained through PLS-DA for discrimination between control (healthy olives) and isolated defects. Values are expressed in number of samples.

Predicted Class			
Real Class	Extra	1st Category	2nd Category
Extra	18	0	0
1st Category	0	45	0
2nd Category	0	0	18

## Data Availability

All relevant data are included within the manuscript. The raw data are available on request from the authors.

## References

[1] Ministerio de Agricultura pesca y Alimentación Aceituna de Mesa. https://www.mapa.gob.es/es/agricultura/temas/producciones-agricolas/aceite-oliva-y-aceituna-mesa/aceituna.aspx.

[2] Royal Degree 679/2016, Norma de Calidad de Las Aceitunas de Mesa. Boletín Oficial del Estado. https://www.boe.es/buscar/act.php?id=BOE-A-2016-11953.

[3] IOC (2021). Method for the Sensory Analysis of Table Olives COI/OT/MO/Doc. No 1/Rev.3.

[4] Lanza B., Amoruso F. (2016). Sensory Analysis of Natural Table Olives: Relationship between Appearance of Defect and Gustatory-Kinaesthetic Sensation Changes. LWT Food Sci. Technol..

[5] Martín-Vertedor D., Schaide T., Boselli E., Martínez M., Arias-Calderón R., Pérez-Nevado F. (2021). Effects of Different Controlled Temperatures on Spanish-Style Fermentation Processes of Olives. Foods.

[6] Lodolini E.M., Cabrera-Bañegil M., Fernández A., Delgado-Adámez J., Ramírez R., Martín-Vertedor D. (2019). Monitoring of Acrylamide and Phenolic Compounds in Table Olive after High Hydrostatic Pressure and Cooking Treatments. Food Chem..

[7] López-López A., Sánchez-Gómez A.H., Montaño A., Cortés-Delgado A., Garrido-Fernández A. (2019). Sensory Characterisation of Black Ripe Table Olives from Spanish Manzanilla and Hojiblanca Cultivars. Food Res. Int..

[8] Panagou E.Z., Sahgal N., Magan N., Nychas G.J.E. (2008). Table Olives Volatile Fingerprints: Potential of an Electronic Nose for Quality Discrimination. Sens. Actuators B Chem..

[9] Xu J., Liu K., Zhang C. (2021). Electronic Nose for Volatile Organic Compounds Analysis in Rice Aging. Trends Food Sci. Technol..

[10] Martínez-García R., Moreno J., Bellincontro A., Centioni L., Puig-Pujol A., Peinado R.A., Mauricio J.C., García-Martínez T. (2021). Using an Electronic Nose and Volatilome Analysis to Differentiate Sparkling Wines Obtained under Different Conditions of Temperature, Ageing Time and Yeast Formats. Food Chem..

[11] Zhang L., Hu Y., Wang Y., Kong B., Chen Q. (2021). Evaluation of the Flavour Properties of Cooked Chicken Drumsticks as Affected by Sugar Smoking Times Using an Electronic Nose, Electronic Tongue, and HS-SPME/GC-MS. LWT.

[12] Majchrzak T., Wojnowski W., Dymerski T., Gębicki J., Namieśnik J. (2018). Electronic Noses in Classification and Quality Control of Edible Oils: A Review. Food Chem..

[13] Arroyo P., Meléndez F., Suárez J.I., Herrero J.L., Rodríguez S., Lozano J. (2020). Electronic Nose with Digital Gas Sensors Connected via Bluetooth to a Smartphone for Air Quality Measurements. Sensors.

[14] Martínez Gila D.M., Gámez García J., Bellincontro A., Mencarelli F., Gómez Ortega J. (2020). Fast Tool Based on Electronic Nose to Predict Olive Fruit Quality after Harvest. Postharvest Biol. Technol..

[15] Schaide T., Cabrera-Bañegil M., Pérez-Nevado F., Esperilla A., Martín-Vertedor D. (2019). Effect of Olive Leaf Extract Combined with Saccharomyces Cerevisiae in the Fermentation Process of Table Olives. J. Food Sci. Technol..

[16] Portalo-Calero F., Arroyo P., Suárez J.I., Lozano J. (2019). Triangular Test of Amanita Mushrooms by Using Electronic Nose and Sensory Panel. Foods.

[17] Arroyo P., Lozano J., Suárez J.I. (2018). Evolution of Wireless Sensor Network for Air Quality Measurements. Electronics.

[18] Barker M., Rayens W. (2003). Partial Least Squares for Discrimination. J. Chemom..

[19] Lanza B. (2013). Abnormal fermentations in table-olive processing: Microbial origin and sensory evaluation. Front. Microbiol..

[20] Marx Í.M.G., Rodrigues N., Dias L.G., Veloso A.C.A., Pereira J.A., Drunkler D.A., Peres A.M. (2017). Quantification of Table Olives’ Acid, Bitter and Salty Tastes Using Potentiometric Electronic Tongue Fingerprints. LWT Food Sci. Technol..

[21] Mas A., Guillamon J.M., Torija M.J., Beltran G., Cerezo A.B., Troncoso A.M., Garcia-Parrilla M.C. (2014). Bioactive Compounds Derived from the Yeast Metabolism of Aromatic Amino Acids during Alcoholic Fermentation. BioMed Res. Int..

[22] Fischer T., Pietruszka J. (2010). Key Building Blocks via Enzyme-Mediated Synthesis. Top. Curr. Chem..

[23] Wang B., Sun M., Yang J., Shen Z., Ou Y., Fu L., Zhao Y., Li R., Ruan Y., Shen Q. (2022). Inducing Banana Fusarium Wilt Disease Suppression through Soil Microbiome Reshaping by Pineapple-Banana Rotation Combined with Biofertilizer Application. Soil.

[24] Montaño A., de Castro A., Rejano L., Sánchez A.H. (1992). Analysis of Zapatera Olives by Gas and High-Performance Liquid Chromatography. J. Chromatogr. A.

[25] de Castro A., Sánchez A.H., López-López A., Cortés-Delgado A., Medina E., Montaño A. (2018). Microbiota and Metabolite Profiling of Spoiled Spanish-Style Green Table Olives. Metabolites.

[26] Sansone-Land A., Takeoka G.R., Shoemaker C.F. (2014). Volatile Constituents of Commercial Imported and Domestic Black-Ripe Table Olives (*Olea europaea*). Food Chem..

[27] Liu A., Zhang H., Liu T., Gong P., Wang Y., Wang H., Tian X., Liu Q., Cui Q., Xie X. (2022). Aroma Classification and Flavor Characterization of Streptococcus Thermophilus Fermented Milk by HS-GC-IMS and HS-SPME-GC-TOF/MS. Food Biosci..

[28] Sánchez R., Martín-tornero E., Lozano J., Boselli E., Arroyo P., Meléndez F., Martín-vertedor D. (2021). E-nose Discrimination of Abnormal Fermentations in Spanish-style Green Olives. Molecules.

[29] Sánchez R., Pérez-Nevado F., Montero-Fernández I., Lozano J., Meléndez F., Martín-Vertedor D. (2022). Application of Electronic Nose to Discriminate Species of Mold Strains in Synthetic Brines. Front. Microbiol..

[30] Mota I., Teixeira-Santos R., Cavaleiro Rufo J. (2021). Detection and Identification of Fungal Species by Electronic Nose Technology: A Systematic Review. Fungal Biol. Rev..

[31] Loulier J., Lefort F., Stocki M., Asztemborska M., Szmigielski R., Siwek K., Grzywacz T., Hsiang T., Ślusarski S., Oszako T. (2020). Detection of Fungi and Oomycetes by Volatiles Using E-Nose and Spme-Gc/Ms Platforms. Molecules.

[32] Sánchez R., Martín-Tornero E., Lozano J., Fernández A., Arroyo P., Meléndez F., Martín-Vertedor D. (2022). Electronic nose application for the discrimination of sterilization treatments applied to Californian-style black olive varieties. J. Sci. Food Agric..

[33] Sánchez R., Martín-Tornero E., Lozano J., Arroyo P., Meléndez F., Martín-Vertedor D. (2022). Evaluation of the olfactory pattern of black olives stuffed with flavored hydrocolloids. LWT.

[34] Sánchez R., Boselli E., Fernández A., Arroyo P., Lozano J., Martín-Vertedor D. (2022). Determination of the Masking Effect of the ‘Zapateria’Defect in Flavoured Stuffed Olives Using E-Nose. Molecules.

